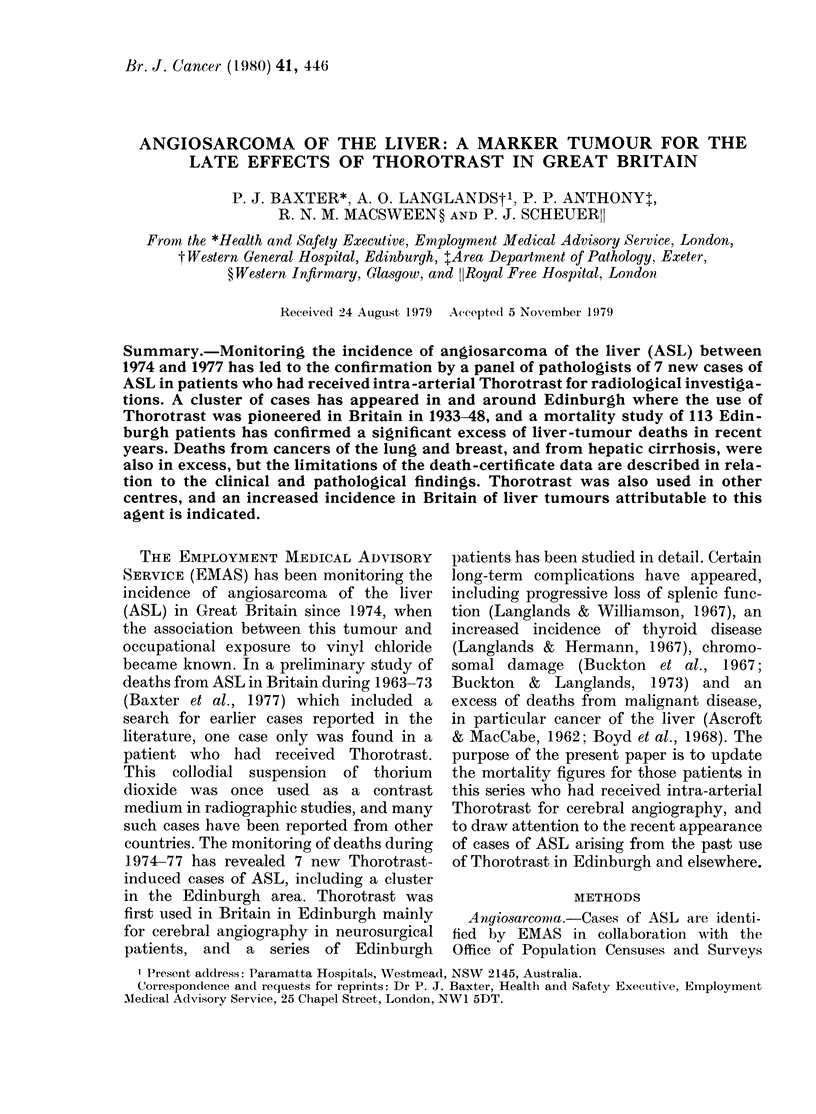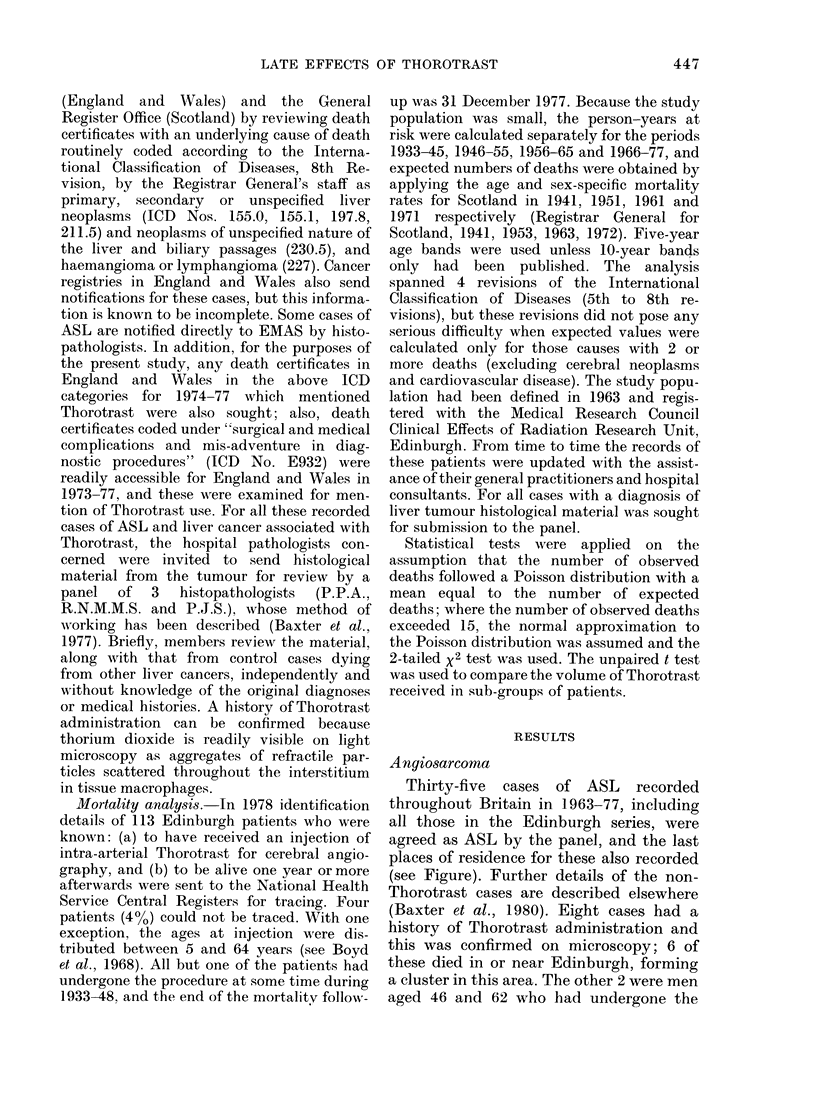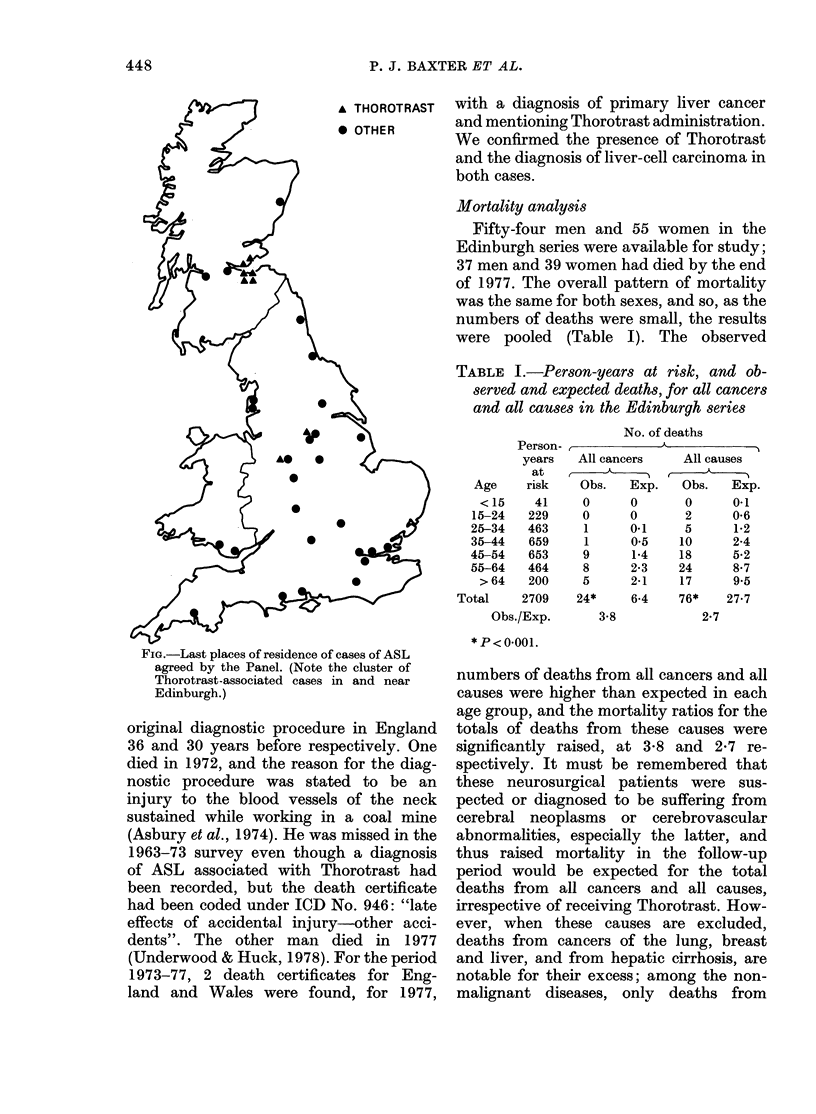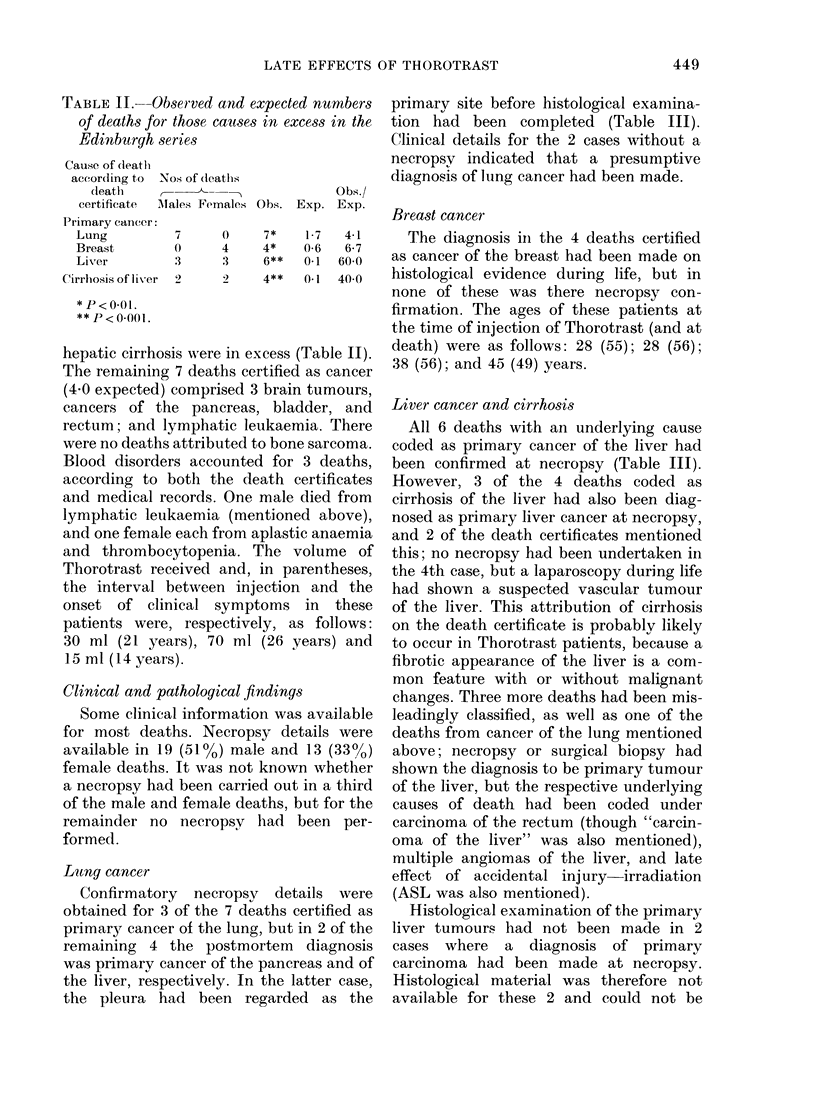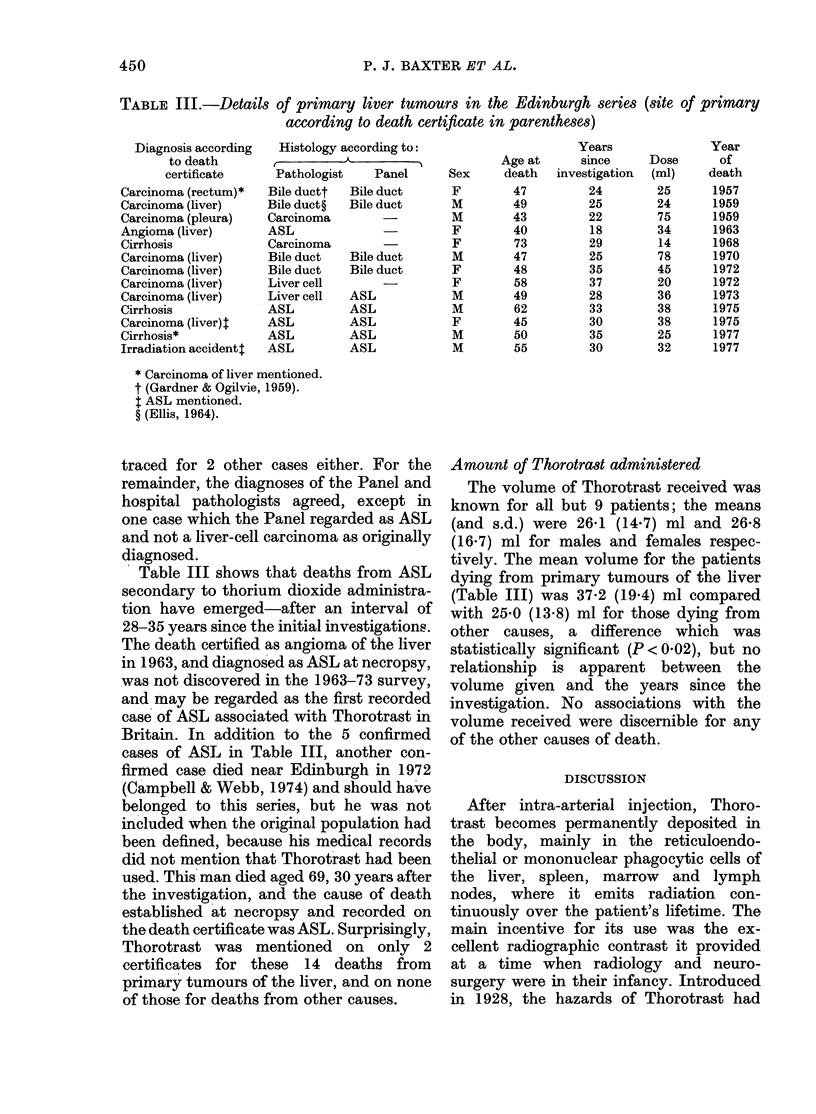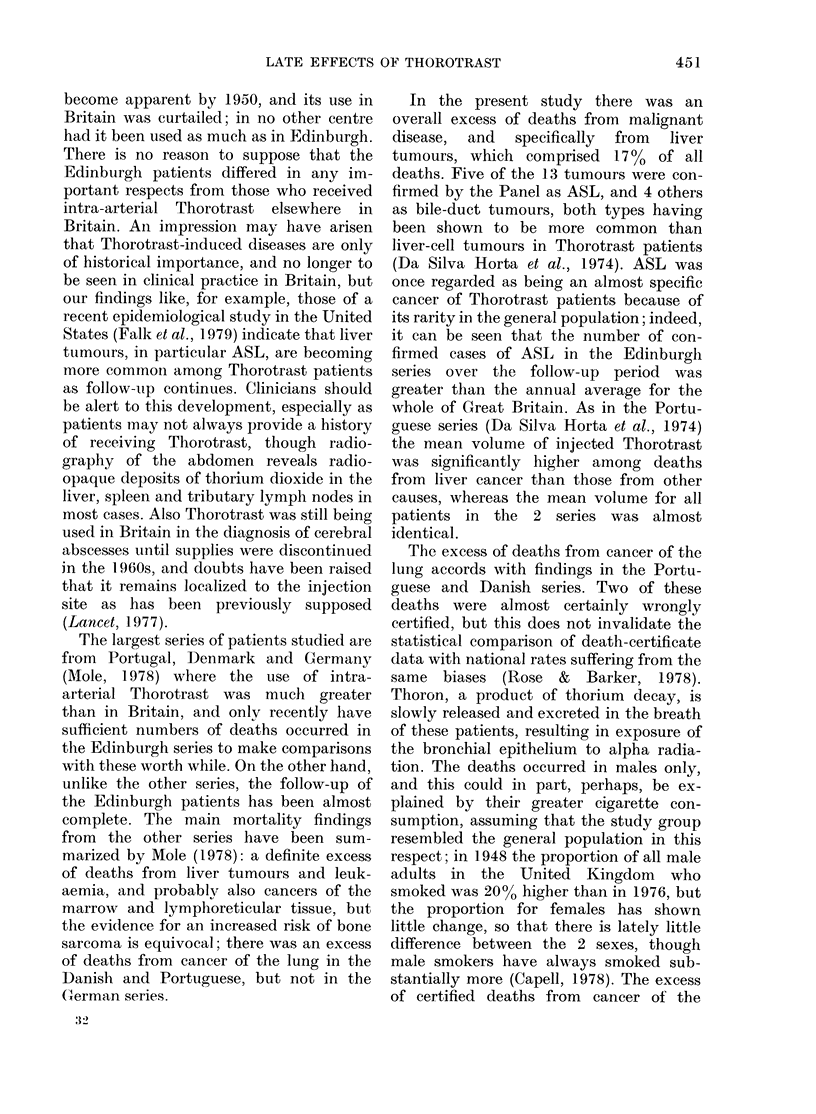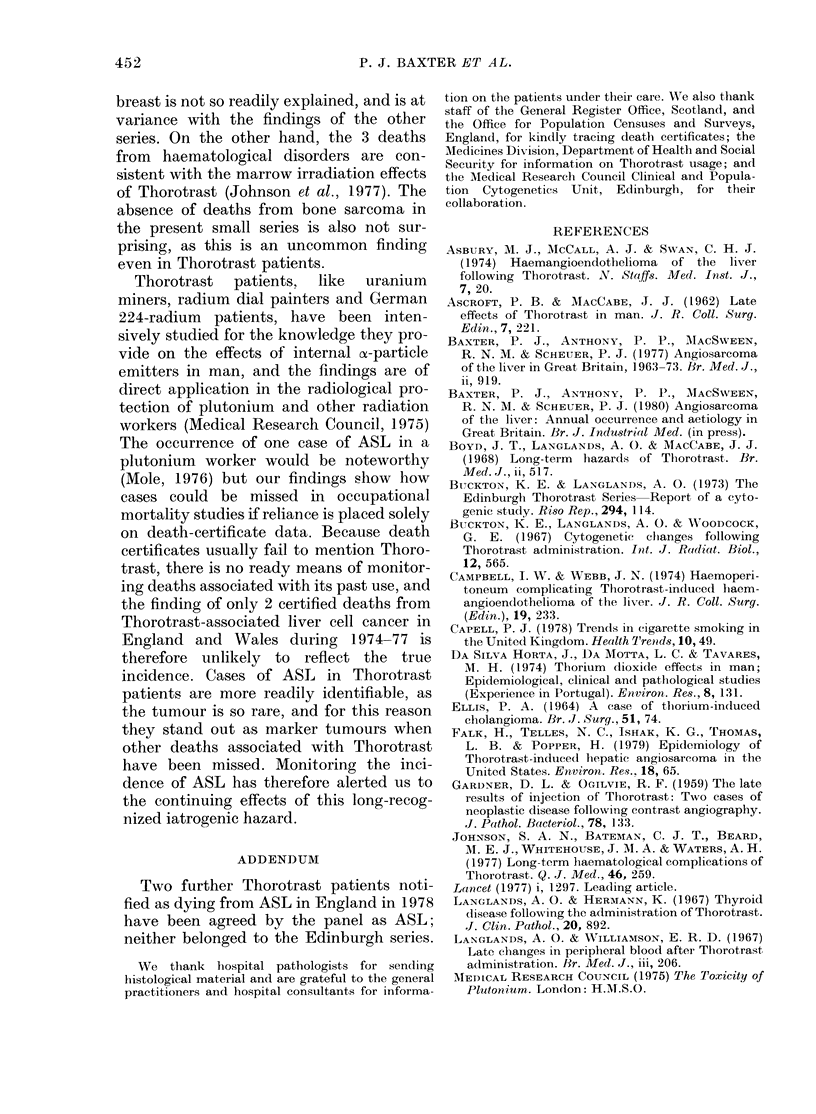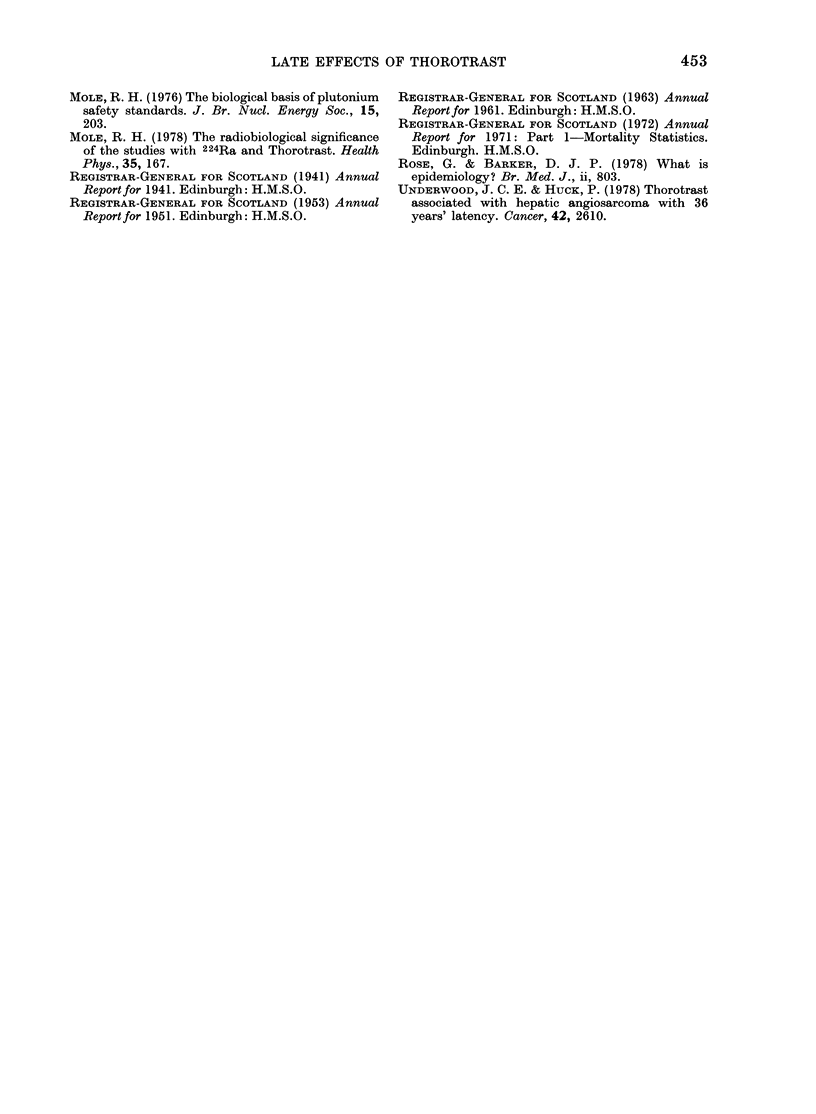# Angiosarcoma of the liver: a marker tumour for the late effects of Thorotrast in Great Britain.

**DOI:** 10.1038/bjc.1980.69

**Published:** 1980-03

**Authors:** P. J. Baxter, A. O. Langlands, P. P. Anthony, R. N. Macsween, P. J. Scheuer

## Abstract

Monitoring the incidence of angiosarcoma of the liver (ASL) between 1974 and 1977 has led to the confirmation by a panel of pathologists of 7 new cases of ASL in patients who had received intra-arterial Thorotrast for radiological investigations. A cluster of cases has appeared in and around Edinburgh where the use of Thorotrast was pioneered in Britain in 1933-48, and a mortality study of 113 Edinburgg patients has confirmed a significant excess of liver-tumour deaths in recent years. Deaths from cancers of the lung and breast, and from hepatic cirrhosis, were also in excess, but the limitations of the death-certificate data are described in relation to the clinical and pathological findings. Thorotrast was also used in other centres, and an increased incidence in Britain of liver tumours attributable to this agent is indicated.


Br. J. Cancer (1980) 41, 446

### ANGIOSARCOMA OF THE LIVER: A MARKER TUMOUR FOR THE LATE EFFECTS OF THOROTRAST IN GREAT BRITAIN

P. J. BAXTER*, A. O. LANGLANDS†[1], P. P. ANTHONY‡, R. N. M. MACSWEEN§ AND P. J. SCHEUER||

*From the *Health and Safety Executive, Employment Medical Advisory Service, London, †Western General Hospital, Edinburgh, ‡Area Department of Pathology, Exeter, §Western Infirmary, Glasgow, and ||Royal Free Hospital, London*

Received 24 August 1979 Accepted 5 November 1979

**Summary.—Monitoring the incidence of angiosarcoma of the liver (ASL) between 1974 and 1977 has led to the confirmation by a panel of pathologists of 7 new cases of ASL in patients who had received intra-arterial Thorotrast for radiological investigations. A cluster of cases has appeared in and around Edinburgh where the use of Thorotrast was pioneered in Britain in 1933–48, and a mortality study of 113 Edinburgh patients has confirmed a significant excess of liver-tumour deaths in recent years. Deaths from cancers of the lung and breast, and from hepatic cirrhosis, were also in excess, but the limitations of the death-certificate data are described in relation to the clinical and pathological findings. Thorotrast was also used in other centres, and an increased incidence in Britain of liver tumours attributable to this agent is indicated.**

THE EMPLOYMENT MEDICAL ADVISORY SERVICE (EMAS) has been monitoring the incidence of angiosarcoma of the liver (ASL) in Great Britain since 1974, when the association between this tumour and occupational exposure to vinyl chloride became known. In a preliminary study of deaths from ASL in Britain during 1963–73 (Baxter *et al.*, 1977) which included a search for earlier cases reported in the literature, one case only was found in a patient who had received Thorotrast. This collodial suspension of thorium dioxide was once used as a contrast medium in radiographic studies, and many such cases have been reported from other countries. The monitoring of deaths during 1974–77 has revealed 7 new Thorotrast-induced cases of ASL, including a cluster in the Edinburgh area. Thorotrast was first used in Britain in Edinburgh mainly for cerebral angiography in neurosurgical patients, and a series of Edinburgh patients has been studied in detail. Certain long-term complications have appeared, including progressive loss of splenic function (Langlands & Williamson, 1967), an increased incidence of thyroid disease (Langlands & Hermann, 1967), chromosomal damage (Buckton *et al.*, 1967; Buckton & Langlands, 1973) and an excess of deaths from malignant disease, in particular cancer of the liver (Ascroft & MacCabe, 1962; Boyd *et al.*, 1968). The purpose of the present paper is to update the mortality figures for those patients in this series who had received intra-arterial Thorotrast for cerebral angiography, and to draw attention to the recent appearance of cases of ASL arising from the past use of Thorotrast in Edinburgh and elsewhere.

### METHODS

*Angiosarcoma.*—Cases of ASL are identified by EMAS in collaboration with the Office of Population Censuses and Surveys

[1] Present address: Paramatta Hospitals, Westmead, NSW 2145, Australia.
Correspondence and requests for reprints: Dr P. J. Baxter, Health and Safety Executive, Employment Medical Advisory Service, 25 Chapel Street, London, NW1 5DT.

(England and Wales) and the General Register Office (Scotland) by reviewing death certificates with an underlying cause of death routinely coded according to the International Classification of Diseases, 8th Revision, by the Registrar General's staff as primary, secondary or unspecified liver neoplasms (ICD Nos. 155.0, 155.1, 197.8, 211.5) and neoplasms of unspecified nature of the liver and biliary passages (230.5), and haemangioma or lymphangioma (227). Cancer registries in England and Wales also send notifications for these cases, but this information is known to be incomplete. Some cases of ASL are notified directly to EMAS by histopathologists. In addition, for the purposes of the present study, any death certificates in England and Wales in the above ICD categories for 1974–77 which mentioned Thorotrast were also sought; also, death certificates coded under "surgical and medical complications and mis-adventure in diagnostic procedures" (ICD No. E932) were readily accessible for England and Wales in 1973–77, and these were examined for mention of Thorotrast use. For all these recorded cases of ASL and liver cancer associated with Thorotrast, the hospital pathologists concerned were invited to send histological material from the tumour for review by a panel of 3 histopathologists (P.P.A., R.N.M.M.S. and P.J.S.), whose method of working has been described (Baxter *et al.*, 1977). Briefly, members review the material, along with that from control cases dying from other liver cancers, independently and without knowledge of the original diagnoses or medical histories. A history of Thorotrast administration can be confirmed because thorium dioxide is readily visible on light microscopy as aggregates of refractile particles scattered throughout the interstitium in tissue macrophages.

*Mortality analysis.*—In 1978 identification details of 113 Edinburgh patients who were known: (a) to have received an injection of intra-arterial Thorotrast for cerebral angiography, and (b) to be alive one year or more afterwards were sent to the National Health Service Central Registers for tracing. Four patients (4%) could not be traced. With one exception, the ages at injection were distributed between 5 and 64 years (see Boyd *et al.*, 1968). All but one of the patients had undergone the procedure at some time during 1933–48, and the end of the mortality follow-up was 31 December 1977. Because the study population was small, the person–years at risk were calculated separately for the periods 1933–45, 1946–55, 1956–65 and 1966–77, and expected numbers of deaths were obtained by applying the age and sex-specific mortality rates for Scotland in 1941, 1951, 1961 and 1971 respectively (Registrar General for Scotland, 1941, 1953, 1963, 1972). Five-year age bands were used unless 10-year bands only had been published. The analysis spanned 4 revisions of the International Classification of Diseases (5th to 8th revisions), but these revisions did not pose any serious difficulty when expected values were calculated only for those causes with 2 or more deaths (excluding cerebral neoplasms and cardiovascular disease). The study population had been defined in 1963 and registered with the Medical Research Council Clinical Effects of Radiation Research Unit, Edinburgh. From time to time the records of these patients were updated with the assistance of their general practitioners and hospital consultants. For all cases with a diagnosis of liver tumour histological material was sought for submission to the panel.

Statistical tests were applied on the assumption that the number of observed deaths followed a Poisson distribution with a mean equal to the number of expected deaths; where the number of observed deaths exceeded 15, the normal approximation to the Poisson distribution was assumed and the 2-tailed $\chi^2$ test was used. The unpaired $t$ test was used to compare the volume of Thorotrast received in sub-groups of patients.

### RESULTS

### *Angiosarcoma*

Thirty-five cases of ASL recorded throughout Britain in 1963–77, including all those in the Edinburgh series, were agreed as ASL by the panel, and the last places of residence for these also recorded (see Figure). Further details of the non-Thorotrast cases are described elsewhere (Baxter *et al.*, 1980). Eight cases had a history of Thorotrast administration and this was confirmed on microscopy; 6 of these died in or near Edinburgh, forming a cluster in this area. The other 2 were men aged 46 and 62 who had undergone the

FIG.—Last places of residence of cases of ASL agreed by the Panel. (Note the cluster of Thorotrast-associated cases in and near Edinburgh.)

original diagnostic procedure in England 36 and 30 years before respectively. One died in 1972, and the reason for the diagnostic procedure was stated to be an injury to the blood vessels of the neck sustained while working in a coal mine (Asbury *et al.*, 1974). He was missed in the 1963–73 survey even though a diagnosis of ASL associated with Thorotrast had been recorded, but the death certificate had been coded under ICD No. 946: "late effects of accidental injury—other accidents". The other man died in 1977 (Underwood & Huck, 1978). For the period 1973–77, 2 death certificates for England and Wales were found, for 1977, with a diagnosis of primary liver cancer and mentioning Thorotrast administration. We confirmed the presence of Thorotrast and the diagnosis of liver-cell carcinoma in both cases.

*Mortality analysis*

Fifty-four men and 55 women in the Edinburgh series were available for study; 37 men and 39 women had died by the end of 1977. The overall pattern of mortality was the same for both sexes, and so, as the numbers of deaths were small, the results were pooled (Table I). The observed

TABLE I.—*Person-years at risk, and observed and expected deaths, for all cancers and all causes in the Edinburgh series*

| | | No. of deaths | | | |
|---|---|---|---|---|---|
| Age | Person-years at risk | All cancers Obs. | All cancers Exp. | All causes Obs. | All causes Exp. |
| $<15$ | 41 | 0 | 0 | 0 | 0·1 |
| 15–24 | 229 | 0 | 0 | 2 | 0·6 |
| 25–34 | 463 | 1 | 0·1 | 5 | 1·2 |
| 35–44 | 659 | 1 | 0·5 | 10 | 2·4 |
| 45–54 | 653 | 9 | 1·4 | 18 | 5·2 |
| 55–64 | 464 | 8 | 2·3 | 24 | 8·7 |
| $>64$ | 200 | 5 | 2·1 | 17 | 9·5 |
| Total | 2709 | 24* | 6·4 | 76* | 27·7 |
| Obs./Exp. | | 3·8 | | 2·7 | |

* $P < 0·001$.

numbers of deaths from all cancers and all causes were higher than expected in each age group, and the mortality ratios for the totals of deaths from these causes were significantly raised, at 3·8 and 2·7 respectively. It must be remembered that these neurosurgical patients were suspected or diagnosed to be suffering from cerebral neoplasms or cerebrovascular abnormalities, especially the latter, and thus raised mortality in the follow-up period would be expected for the total deaths from all cancers and all causes, irrespective of receiving Thorotrast. However, when these causes are excluded, deaths from cancers of the lung, breast and liver, and from hepatic cirrhosis, are notable for their excess; among the non-malignant diseases, only deaths from

TABLE II.—*Observed and expected numbers of deaths for those causes in excess in the Edinburgh series*

| Cause of death according to death certificate | Nos of deaths Males | Females | Obs. | Exp. | Obs./Exp. |
|---|---|---|---|---|---|
| Primary cancer: | | | | | |
| Lung | 7 | 0 | 7* | 1·7 | 4·1 |
| Breast | 0 | 4 | 4* | 0·6 | 6·7 |
| Liver | 3 | 3 | 6** | 0·1 | 60·0 |
| Cirrhosis of liver | 2 | 2 | 4** | 0·1 | 40·0 |

* $P < 0{\cdot}01$.
** $P < 0{\cdot}001$.

hepatic cirrhosis were in excess (Table II). The remaining 7 deaths certified as cancer (4·0 expected) comprised 3 brain tumours, cancers of the pancreas, bladder, and rectum; and lymphatic leukaemia. There were no deaths attributed to bone sarcoma. Blood disorders accounted for 3 deaths, according to both the death certificates and medical records. One male died from lymphatic leukaemia (mentioned above), and one female each from aplastic anaemia and thrombocytopenia. The volume of Thorotrast received and, in parentheses, the interval between injection and the onset of clinical symptoms in these patients were, respectively, as follows: 30 ml (21 years), 70 ml (26 years) and 15 ml (14 years).

*Clinical and pathological findings*

Some clinical information was available for most deaths. Necropsy details were available in 19 (51%) male and 13 (33%) female deaths. It was not known whether a necropsy had been carried out in a third of the male and female deaths, but for the remainder no necropsy had been performed.

*Lung cancer*

Confirmatory necropsy details were obtained for 3 of the 7 deaths certified as primary cancer of the lung, but in 2 of the remaining 4 the postmortem diagnosis was primary cancer of the pancreas and of the liver, respectively. In the latter case, the pleura had been regarded as the primary site before histological examination had been completed (Table III). Clinical details for the 2 cases without a necropsy indicated that a presumptive diagnosis of lung cancer had been made.

*Breast cancer*

The diagnosis in the 4 deaths certified as cancer of the breast had been made on histological evidence during life, but in none of these was there necropsy confirmation. The ages of these patients at the time of injection of Thorotrast (and at death) were as follows: 28 (55); 28 (56); 38 (56); and 45 (49) years.

*Liver cancer and cirrhosis*

All 6 deaths with an underlying cause coded as primary cancer of the liver had been confirmed at necropsy (Table III). However, 3 of the 4 deaths coded as cirrhosis of the liver had also been diagnosed as primary liver cancer at necropsy, and 2 of the death certificates mentioned this; no necropsy had been undertaken in the 4th case, but a laparoscopy during life had shown a suspected vascular tumour of the liver. This attribution of cirrhosis on the death certificate is probably likely to occur in Thorotrast patients, because a fibrotic appearance of the liver is a common feature with or without malignant changes. Three more deaths had been misleadingly classified, as well as one of the deaths from cancer of the lung mentioned above; necropsy or surgical biopsy had shown the diagnosis to be primary tumour of the liver, but the respective underlying causes of death had been coded under carcinoma of the rectum (though "carcinoma of the liver" was also mentioned), multiple angiomas of the liver, and late effect of accidental injury—irradiation (ASL was also mentioned).

Histological examination of the primary liver tumours had not been made in 2 cases where a diagnosis of primary carcinoma had been made at necropsy. Histological material was therefore not available for these 2 and could not be

TABLE III.—*Details of primary liver tumours in the Edinburgh series (site of primary according to death certificate in parentheses)*

| Diagnosis according to death certificate | Histology according to: Pathologist | Panel | Sex | Age at death | Years since investigation | Dose (ml) | Year of death |
|---|---|---|---|---|---|---|---|
| Carcinoma (rectum)* | Bile duct† | Bile duct | F | 47 | 24 | 25 | 1957 |
| Carcinoma (liver) | Bile duct§ | Bile duct | M | 49 | 25 | 24 | 1959 |
| Carcinoma (pleura) | Carcinoma | — | M | 43 | 22 | 75 | 1959 |
| Angioma (liver) | ASL | — | F | 40 | 18 | 34 | 1963 |
| Cirrhosis | Carcinoma | — | F | 73 | 29 | 14 | 1968 |
| Carcinoma (liver) | Bile duct | Bile duct | M | 47 | 25 | 78 | 1970 |
| Carcinoma (liver) | Bile duct | Bile duct | F | 48 | 35 | 45 | 1972 |
| Carcinoma (liver) | Liver cell | — | F | 58 | 37 | 20 | 1972 |
| Carcinoma (liver) | Liver cell | ASL | M | 49 | 28 | 36 | 1973 |
| Cirrhosis | ASL | ASL | M | 62 | 33 | 38 | 1975 |
| Carcinoma (liver)‡ | ASL | ASL | F | 45 | 30 | 38 | 1975 |
| Cirrhosis* | ASL | ASL | M | 50 | 35 | 25 | 1977 |
| Irradiation accident‡ | ASL | ASL | M | 55 | 30 | 32 | 1977 |

* Carcinoma of liver mentioned.

† (Gardner & Ogilvie, 1959).

‡ ASL mentioned.

§ (Ellis, 1964).

traced for 2 other cases either. For the remainder, the diagnoses of the Panel and hospital pathologists agreed, except in one case which the Panel regarded as ASL and not a liver-cell carcinoma as originally diagnosed.

Table III shows that deaths from ASL secondary to thorium dioxide administration have emerged—after an interval of 28–35 years since the initial investigations. The death certified as angioma of the liver in 1963, and diagnosed as ASL at necropsy, was not discovered in the 1963–73 survey, and may be regarded as the first recorded case of ASL associated with Thorotrast in Britain. In addition to the 5 confirmed cases of ASL in Table III, another confirmed case died near Edinburgh in 1972 (Campbell & Webb, 1974) and should have belonged to this series, but he was not included when the original population had been defined, because his medical records did not mention that Thorotrast had been used. This man died aged 69, 30 years after the investigation, and the cause of death established at necropsy and recorded on the death certificate was ASL. Surprisingly, Thorotrast was mentioned on only 2 certificates for these 14 deaths from primary tumours of the liver, and on none of those for deaths from other causes.

*Amount of Thorotrast administered*

The volume of Thorotrast received was known for all but 9 patients; the means (and s.d.) were 26·1 (14·7) ml and 26·8 (16·7) ml for males and females respectively. The mean volume for the patients dying from primary tumours of the liver (Table III) was 37·2 (19·4) ml compared with 25·0 (13·8) ml for those dying from other causes, a difference which was statistically significant ($P < 0{\cdot}02$), but no relationship is apparent between the volume given and the years since the investigation. No associations with the volume received were discernible for any of the other causes of death.

### DISCUSSION

After intra-arterial injection, Thorotrast becomes permanently deposited in the body, mainly in the reticuloendothelial or mononuclear phagocytic cells of the liver, spleen, marrow and lymph nodes, where it emits radiation continuously over the patient's lifetime. The main incentive for its use was the excellent radiographic contrast it provided at a time when radiology and neurosurgery were in their infancy. Introduced in 1928, the hazards of Thorotrast had become apparent by 1950, and its use in Britain was curtailed; in no other centre had it been used as much as in Edinburgh. There is no reason to suppose that the Edinburgh patients differed in any important respects from those who received intra-arterial Thorotrast elsewhere in Britain. An impression may have arisen that Thorotrast-induced diseases are only of historical importance, and no longer to be seen in clinical practice in Britain, but our findings like, for example, those of a recent epidemiological study in the United States (Falk *et al.*, 1979) indicate that liver tumours, in particular ASL, are becoming more common among Thorotrast patients as follow-up continues. Clinicians should be alert to this development, especially as patients may not always provide a history of receiving Thorotrast, though radiography of the abdomen reveals radio-opaque deposits of thorium dioxide in the liver, spleen and tributary lymph nodes in most cases. Also Thorotrast was still being used in Britain in the diagnosis of cerebral abscesses until supplies were discontinued in the 1960s, and doubts have been raised that it remains localized to the injection site as has been previously supposed (*Lancet*, 1977).

The largest series of patients studied are from Portugal, Denmark and Germany (Mole, 1978) where the use of intra-arterial Thorotrast was much greater than in Britain, and only recently have sufficient numbers of deaths occurred in the Edinburgh series to make comparisons with these worth while. On the other hand, unlike the other series, the follow-up of the Edinburgh patients has been almost complete. The main mortality findings from the other series have been summarized by Mole (1978): a definite excess of deaths from liver tumours and leukaemia, and probably also cancers of the marrow and lymphoreticular tissue, but the evidence for an increased risk of bone sarcoma is equivocal; there was an excess of deaths from cancer of the lung in the Danish and Portuguese, but not in the German series.

In the present study there was an overall excess of deaths from malignant disease, and specifically from liver tumours, which comprised 17% of all deaths. Five of the 13 tumours were confirmed by the Panel as ASL, and 4 others as bile-duct tumours, both types having been shown to be more common than liver-cell tumours in Thorotrast patients (Da Silva Horta *et al.*, 1974). ASL was once regarded as being an almost specific cancer of Thorotrast patients because of its rarity in the general population; indeed, it can be seen that the number of confirmed cases of ASL in the Edinburgh series over the follow-up period was greater than the annual average for the whole of Great Britain. As in the Portuguese series (Da Silva Horta *et al.*, 1974) the mean volume of injected Thorotrast was significantly higher among deaths from liver cancer than those from other causes, whereas the mean volume for all patients in the 2 series was almost identical.

The excess of deaths from cancer of the lung accords with findings in the Portuguese and Danish series. Two of these deaths were almost certainly wrongly certified, but this does not invalidate the statistical comparison of death-certificate data with national rates suffering from the same biases (Rose & Barker, 1978). Thoron, a product of thorium decay, is slowly released and excreted in the breath of these patients, resulting in exposure of the bronchial epithelium to alpha radiation. The deaths occurred in males only, and this could in part, perhaps, be explained by their greater cigarette consumption, assuming that the study group resembled the general population in this respect; in 1948 the proportion of all male adults in the United Kingdom who smoked was 20% higher than in 1976, but the proportion for females has shown little change, so that there is lately little difference between the 2 sexes, though male smokers have always smoked substantially more (Capell, 1978). The excess of certified deaths from cancer of the breast is not so readily explained, and is at variance with the findings of the other series. On the other hand, the 3 deaths from haematological disorders are consistent with the marrow irradiation effects of Thorotrast (Johnson *et al.*, 1977). The absence of deaths from bone sarcoma in the present small series is also not surprising, as this is an uncommon finding even in Thorotrast patients.

Thorotrast patients, like uranium miners, radium dial painters and German 224-radium patients, have been intensively studied for the knowledge they provide on the effects of internal $\alpha$-particle emitters in man, and the findings are of direct application in the radiological protection of plutonium and other radiation workers (Medical Research Council, 1975) The occurrence of one case of ASL in a plutonium worker would be noteworthy (Mole, 1976) but our findings show how cases could be missed in occupational mortality studies if reliance is placed solely on death-certificate data. Because death certificates usually fail to mention Thorotrast, there is no ready means of monitoring deaths associated with its past use, and the finding of only 2 certified deaths from Thorotrast-associated liver cell cancer in England and Wales during 1974–77 is therefore unlikely to reflect the true incidence. Cases of ASL in Thorotrast patients are more readily identifiable, as the tumour is so rare, and for this reason they stand out as marker tumours when other deaths associated with Thorotrast have been missed. Monitoring the incidence of ASL has therefore alerted us to the continuing effects of this long-recognized iatrogenic hazard.

### ADDENDUM

Two further Thorotrast patients notified as dying from ASL in England in 1978 have been agreed by the panel as ASL; neither belonged to the Edinburgh series.

We thank hospital pathologists for sending histological material and are grateful to the general practitioners and hospital consultants for information on the patients under their care. We also thank staff of the General Register Office, Scotland, and the Office for Population Censuses and Surveys, England, for kindly tracing death certificates; the Medicines Division, Department of Health and Social Security for information on Thorotrast usage; and the Medical Research Council Clinical and Population Cytogenetics Unit, Edinburgh, for their collaboration.

### REFERENCES

Asbury, M. J., McCall, A. J. & Swan, C. H. J. (1974) Haemangioendothelioma of the liver following Thorotrast. *N. Staffs. Med. Inst. J.*, **7**, 20.

Ascroft, P. B. & MacCabe, J. J. (1962) Late effects of Thorotrast in man. *J. R. Coll. Surg. Edin.*, **7**, 221.

Baxter, P. J., Anthony, P. P., MacSween, R. N. M. & Scheuer, P. J. (1977) Angiosarcoma of the liver in Great Britain, 1963–73. *Br. Med. J.*, ii, 919.

Baxter, P. J., Anthony, P. P., MacSween, R. N. M. & Scheuer, P. J. (1980) Angiosarcoma of the liver: Annual occurrence and aetiology in Great Britain. *Br. J. Industrial Med.* (in press).

Boyd, J. T., Langlands, A. O. & MacCabe, J. J. (1968) Long-term hazards of Thorotrast. *Br. Med. J.*, ii, 517.

Buckton, K. E. & Langlands, A. O. (1973) The Edinburgh Thorotrast Series—Report of a cytogenic study. *Riso Rep.*, **294**, 114.

Buckton, K. E., Langlands, A. O. & Woodcock, G. E. (1967) Cytogenetic changes following Thorotrast administration. *Int. J. Radiat. Biol.*, **12**, 565.

Campbell, I. W. & Webb, J. N. (1974) Haemoperitoneum complicating Thorotrast-induced haemangioendothelioma of the liver. *J. R. Coll. Surg. (Edin.)*, **19**, 233.

Capell, P. J. (1978) Trends in cigarette smoking in the United Kingdom. *Health Trends*, **10**, 49.

Da Silva Horta, J., Da Motta, L. C. & Tavares, M. H. (1974) Thorium dioxide effects in man; Epidemiological, clinical and pathological studies (Experience in Portugal). *Environ. Res.*, **8**, 131.

Ellis, P. A. (1964) A case of thorium-induced cholangioma. *Br. J. Surg.*, **51**, 74.

Falk, H., Telles, N. C., Ishak, K. G., Thomas, L. B. & Popper, H. (1979) Epidemiology of Thorotrast-induced hepatic angiosarcoma in the United States. *Environ. Res.*, **18**, 65.

Gardner, D. L. & Ogilvie, R. F. (1959) The late results of injection of Thorotrast: Two cases of neoplastic disease following contrast angiography. *J. Pathol. Bacteriol.*, **78**, 133.

Johnson, S. A. N., Bateman, C. J. T., Beard, M. E. J., Whitehouse, J. M. A. & Waters, A. H. (1977) Long-term haematological complications of Thorotrast. *Q. J. Med.*, **46**, 259.

*Lancet* (1977) i, 1297. Leading article.

Langlands, A. O. & Hermann, K. (1967) Thyroid disease following the administration of Thorotrast. *J. Clin. Pathol.*, **20**, 892.

Langlands, A. O. & Williamson, E. R. D. (1967) Late changes in peripheral blood after Thorotrast administration. *Br. Med. J.*, iii, 206.

Medical Research Council (1975) *The Toxicity of Plutonium.* London: H.M.S.O.

MOLE, R. H. (1976) The biological basis of plutonium safety standards. *J. Br. Nucl. Energy Soc.*, **15**, 203.

MOLE, R. H. (1978) The radiobiological significance of the studies with $^{224}$Ra and Thorotrast. *Health Phys.*, **35**, 167.

REGISTRAR-GENERAL FOR SCOTLAND (1941) *Annual Report for* 1941. Edinburgh: H.M.S.O.

REGISTRAR-GENERAL FOR SCOTLAND (1953) *Annual Report for* 1951. Edinburgh: H.M.S.O.

REGISTRAR-GENERAL FOR SCOTLAND (1963) *Annual Report for* 1961. Edinburgh: H.M.S.O.

REGISTRAR-GENERAL FOR SCOTLAND (1972) *Annual Report for* 1971: Part 1—Mortality Statistics. Edinburgh. H.M.S.O.

ROSE, G. & BARKER, D. J. P. (1978) What is epidemiology? *Br. Med. J.*, ii, 803.

UNDERWOOD, J. C. E. & HUCK, P. (1978) Thorotrast associated with hepatic angiosarcoma with 36 years' latency. *Cancer*, **42**, 2610.